# Developing Framework and Strategies for Capacity Building to Apply Evidence-Informed Health Policy-Making in Iran: Mixed Methods Study of SAHSHA Project

**DOI:** 10.34172/ijhpm.2021.142

**Published:** 2021-10-11

**Authors:** Mahdi Mahdavi, Javad Sajjadi Khasraghi, Haniye Sadat Sajadi, Bahareh Yazdizadeh, Sima Nikooee, Elham Ehsani-Chimeh, Hossein Dargahi, Leila Doshmangir, Shahram Ghaffari, Reza Toyserkanmanesh, Reza Majdzadeh

**Affiliations:** ^1^The Bernard Lown Scholar in Cardiovascular Health, Harvard T.H. Chan School of Public Health, Boston, MA, USA.; ^2^National Institute for Health Research, Tehran University of Medical Sciences, Tehran, Iran.; ^3^School of Public Health, Tehran University of Medical Sciences, Tehran, Iran.; ^4^Knowledge Utilization Research Center, University Research and Development Center, Tehran University of Medical Sciences, Tehran, Iran.; ^5^Knowledge Utilization Research Center, Tehran University of Medical Sciences, Tehran, Iran.; ^6^Tabriz Health Services Management Research Center, Department of Health Policy & Management, Tabriz University of Medical Sciences, Tabriz, Iran.; ^7^Social Determinants of Health Research Center, Health Management and Safety Promotion Research Institute, Tabriz University of Medical Sciences, Tabriz, Iran.; ^8^Iranian Social Security Organization, Tehran, Iran.; ^9^Social Security Research Institute, Tehran, Iran.

**Keywords:** Evidence-Informed Policy-Making, Health Policy, Capacity Building Programs, Iran

## Abstract

**Background:** SASHA, which stands for "evidence-informed health policy-making (EIHP)" in Persian, is a national project to draw a roadmap for strengthening EIHP in Iran. As a part of SASHA, this research aimed to develop evidence-based and context-aware policy options for increasing the capacity of decision-makers to apply EIHP in Iran.

**Methods:** This was a qualitative study, which was informed by a literature review of pull efforts’ capacity building programs. Based on the review, we developed policy options and validated them through an expert panel that involved twelve experts. Data were analyzed using a content analysis method.

**Results:** We extracted data from 11 articles. The objectives of capacity building programs were: single-skill development, personal/professional development, and organizational development. According to these objectives, the contents and training methods of the programs vary. Capacity building programs have shown positive impacts on individual knowledge/attitudes to use EIHP. However, the impacts of programs at the organizational or the health system level remain under-researched. We followed several threads from the literature review through to the expert panel that included training the management team, instead of training managers, training for problem-solving skills, and designing tailored programs. Barriers of capacity building for EIHP regard the context of the health system (weak accountability and the widespread conflict of interest) and healthcare organizational structures (decision support systems, knowledge management infrastructures, and lack of management team). Experts suggested interventions on the barriers, particularly on resolving the conflict of interests before launching new programs. A proposed framework to increase the capacity of health policy-makers incorporates strategies at three levels: capacity building program, organizational structure, and health system context.

**Conclusion:** To prepare the context of Iranian healthcare organizations for capacity building programs, the conflict of interests needs to be resolved, decision-makers should be made more accountable, and healthcare organizations need to provide more knowledge management infrastructures and decision support systems.

## Background

**Key Messages**

** Implications for policy makers**

- The results of this work could be used to inform progresses for developing locally adapted policy options to increase the capacity of healthcare decision-makers for applying evidence to real-life health policies and management decisions.
- This work defined the characteristics of locally adapted capacity building programs. It also identified prominent dimensions of organizational structure and the wider context of the health systems that need to be considered for developing local policy options for evidence-informed decision-making.
- This work sheds light on the aspects of accountability mechanisms and the conflict of interest as preconditions for effective capacity building programs.
- Policy options need to consider three threads for designing capacity building programs: training management teams instead of only training managers, focusing the training on problem-solving skills, and designing dedicated programs based on the needs of target groups.

** Implications for the public**

Evidence-informed health policy-making (EIHP) is the systematic and transparent process to access and appraise evidence as an input into the decision-making process. Capacity building through training and continuing education is a key strategy to foster EIHP. Capacity building programs that are informed by local needs and contextual factors, may increase skills and confidence of health policy-makers to make decisions that generate better outcomes and serve underprivileged populations. As such, identifying the barriers and facilitators of capacity building programs allows developing effective, context-aware, and problem-based capacity building programs. So far, capacity building programs have shown positive impacts on individual knowledge and attitudes of decision-makers towards the use of EIHP. Yet, fundamental improvements are needed in the way that programs are designed and delivered so that they can improve the functions of healthcare organizations as well as health systems.

During the past decades, there has been a meaningful shift in the view of professionals to the use of research evidence for policy-making. The notion ‘evidence-informed policy-making’ has been considered not only in the healthcare arena but also in social science and business management.^1^ This phenomenon is coupled with a growing belief that evidence-informed health policy-making (EIHP) should result in quality decisions in contemporary healthcare.^2,3^

 EIHP is defined as “an approach to policy decisions that aims to ensure that decision-making is well-informed by the best available research evidence. It is characterized by the systematic and transparent access to, and appraisal of, evidence as an input into the policy-making process.”^4^ Evidence, typically, refers to the scientific outputs of research activities that provide information on the effectiveness of an intervention. Evidence has been viewed in a hierarchy, grading evidence from the results of randomized controlled trials as the gold standard to expert opinion.^5^

 Emerging literature criticizes the appropriateness of the hierarchy of evidence and particularly its gold standard for policy issues.^6^ This criticism can be grasped in the light of the way that ‘policy’ is defined. Policy can be safely defined as a decision that is taken through an authoritative process.^7^ In practice, there are plenty of policy instances for which no evidence of randomized controlled trials exists. In such cases, other forms of knowledge such as stakeholder views and knowledge of front-line workers contribute to making decisions. Still, EIHP provides decision-makers with tools to support them in following systematic and transparent processes to identify and appraise research evidence and to apply appropriate evidence.^4^ For issues surrounded with imperfect evidence, EIHP helps reduce the risk of decision-making when the consequence of the decisions does not meet expectations.^8^

 The scarcity of resources in developing countries leaves no room for health policies that deliver no ideal results. To enhance the effectiveness, efficiency, and equity of the health systems in the low- and middle-income countries, the World Health Organization (WHO) recommended focusing on strategies to get the best available research evidence into policy and practice.^9^ EIHP builds the backbone of the WHO program to strengthening health systems. At the heart of this program lies capacity building efforts to enable decision-makers to appreciate the value of the evidence and to identify, appraise and apply research findings into health policies and practices.^9^

 Capacity is defined as “the ability to carry out stated objectives.”^10^ Based on this, “capacity building” is a systematic process of education and training, human resource development, knowledge management and knowledge networks to develop and continuously improve competencies and capabilities of health personnel, health organization, and health system to identify, appraise, select, and apply best available evidence to improve equity, efficiency, and effectiveness of health services.^11^ Two main types (among others) of capacity building efforts can be distinguished^12^; ‘pull effort’ refers to training or continuing education for decision-makers that are initiated and funded by healthcare organizations to increase the capacity of decision-makers to acquire, access, assess, and apply research evidence. In comparison, push effort refers to training or educations by educational institutions that are provided by these organizations within their educational missions.^12^

 Individual capacity building is widely used to increase the understanding and use (hereafter ‘uptake’) of EIHP among healthcare managers and policy-makers.^13^ At this level, decision-makers are trained to use the tools and techniques of EIHP (for example, SUPPORT tools for systematic review,^14^ policy brief,^15^ policy dialogue,^16^ and problem definition^17^) and gain confidence to apply them in practice. Training is also used to reduce resistance to change as it is one of the main barriers of EIHP.^18^ Training in the form of provision of skills to staff is a bottom-up organizational approach to enhance the capacity of organizations to be effective in their operations.^19^

 Designing capacity building programs is a hefty task requiring a strong evidence base. Previous systematic reviews shed light on certain options for training programs. Haynes et al recommend that tailored workshops conducted with goal-focused mentoring in a collaborative manner are promising interventions for capacity building.^3,13^ Ellen et al report general recommendations in terms of enablers and barriers of pull-type capacity building.^18^ Yet, questions as ‘what program,’ for ‘which target group,’ ‘what objective,’ ‘what contents,’ and ‘what method of training’ remain. Therefore, a full-fledge review of capacity building programs to guide designing new programs is urgently needed.

 The use of evidence is not only a technical exercise but also a political issue. Various institutional and broader contextual factors in the outer setting of organizations impede the use of evidence for decision-making,^20^ which in turn affect the success of capacity building programs. Institutional factors such as democratization and decentralization affect the use of evidence.^21^ Decentralized organizations grant a greater degree of authority for decision-making, but at the same time, take a greater risk of misuse or underuse of evidence.^20^ Schleiff et al reported common enablers and barriers in several countries for strengthening institutional capacity for uptake of evidence that revolve around leadership and political will, infrastructure and access to health data, and the structures and processes of EIHP.^22^ Yet, further research is needed to shed light on enablers and barriers of strengthening EIHP through capacity building programs. In this research, we review the literature to synthesize a taxonomy of pull-type capacity building programs and we propose contextualized strategies to increase the capacity of policy-makers for applying EIHP in Iran.

 A multi-phase national project “Evidence-Informed Health Policy-making” (*Siasatgozari e Agah az SHAvahed ” (SASHA)*)” was funded by the Iranian National Institute for Medical Research Development (NIMAD) to draw a roadmap for strengthening EIHP in Iran.^23^ At the first phase of the SASHA project, the barriers of EIHP were identified, which raised fundamental questions on how to empower decision-makers for applying EIHP in the Iranian health system. In this manuscript, we present a literature review of pull efforts’ capacity building programs that synthesizes evidence on the objectives of capacity building programs, learning contents, training methods, and the impacts of programs on participants and organizations. Subsequently, we report a qualitative study that we conducted to validate policy options for capacity building programs among policy-makers to apply EIHP in Iran. Based on the review and the qualitative study, we developed strategies for pull efforts’ capacity building programs in Iran.

## Methods

 Our research methods comprised a literature review and a qualitative study. We conducted the literature review^24^ to synthesize evidence on the objectives of capacity building programs, learning contents, training methods, and the impacts of programs on participants and organizations. We also organized one policy dialogue to discuss the feasibility, barriers, and enablers of policy options, which were developed based on the review, for the context of the Iranian health system.^25^

###  The Literature Review 

 For the review, we searched literature published before June 2019 on policy makers’ capacity building initiatives. In the context of this article, the objective is to increase the capacity of policy-makers to apply EIHP to the planning, delivery, evaluation, and improvement of health services. Our inclusion criteria are as follows.

- Studies present pull-type capacity building for EIHP. Pull-type capacity building refers to training programs, continuing education, or workshops by healthcare organizations to increase the capacity of decision-makers on EIHP.^12^
- Studies present implemented training, continuing education, fellowships, or other pull-type training with contents to increase the capacity of decision-makers for evidence-informed planning, organization, delivery, and evaluation of health services.
- Studies should present qualitative, quantitative, or mixed-method analyses of capacity building programs.
- Studies should report a detailed description of training objectives, target groups, learning materials and contents, and training methods of capacity building efforts.
- Studies should be in English and be peer-reviewed.

####  Search Strategy

 A generic search strategy and a list of search terms were developed for the entire SASHA project. This strategy was implemented by the SASHA team who searched Scopus and PubMed. A list of search terms used in two databases is given in Table 1.

**Table 1:** Search Strategy and Hits Obtained

	**Search**	**Query**	**Result**
**PubMed**			
	#1	Decision-making, Organizational [Mesh]	10 968
	#2	"Policy Making"[Mesh]	23 819
	#3	(((((policymak*[Title/Abstract]) OR policy mak*[Title/Abstract]) OR policy-mak*[Title/Abstract]) OR decisionmak*[Title/Abstract]) OR decision-mak*[Title/Abstract]) OR decision-mak*[Title/Abstract]	153 980
	#4	#1 OR #2	34 300
	#5	#4 AND #3	5890
	#6	"Evidence-Based Practice"[Mesh]	82 197
	#7	evidence*[Title/Abstract] OR informe*[Title/Abstract]	1 727 853
	#8	#6 AND #7	49 669
	#9	#5 AND #8	565
	#10	"evidence informed policy making"[Title/Abstract]	51
	#11	"evidence based policy making"[Title/Abstract]	136
	#12	#9 OR #10 OR #11	689
	#13	#12 Filters: English	673
**Scopus**			
	#1	(TITLE-ABS-KEY ("evidence based policy making”) OR TITLE-ABS-KEY ( "evidence informed policy making"))	546
	#2	Limit English	528
		Total hits	1201

####  Paper Selection 

**Figure 1:**
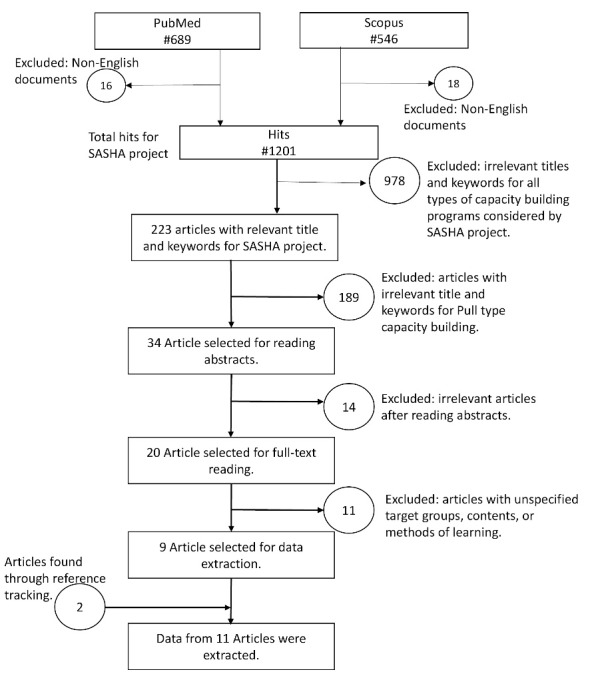
The Article Selection Process.

 We followed a systematic process for paper selection. Two researchers screened titles and keywords to identify relevant studies for all dimensions of the SASHA project that included push, pull, and linkage efforts (Figure 1). The screening resulted in 223 (out of 1201) studies for all dimensions of the SASHA project. Then, two authors of the present research independently screened titles and keywords to identify articles relevant for pull-type capacity building. If both researchers had selected a paper, it was included for data extraction. In case of doubt, the decision was taken based on the full text. In the end, 20 articles were selected for full-text reading, out of which 9 articles were selected for data extraction. Two other articles were identified through backward reference tracking.

####  Data Extraction

 We extracted data from included papers using a data extraction form and on the following dimensions of programs.^26^

1. The objective(s) of capacity building programs in terms of, for instance, increase in knowledge of evidence-informed policy-making.
2. Learning materials, modules, or packages that are presented in a program.
3. Target groups, including policy-makers or healthcare managers at a strategic, tactical, or operational level, who have participated in programs.
4. Learning methods, describing how programs have been implemented; learning methods (virtual class or classroom/workshop) to enhance knowledge or applied skills.
5. Evaluating whether and how improvements in knowledge, attitude, and/or practice of participants were assessed.
6. Participant selection, describing whether programs’ participants were selected through a competitive application or by invitation.
7. Program duration, indicating the length of time that takes from onset to complete the program.

####  Synthesis Methods

 We relied on a narrative summary of literature for synthesizing data extracted from the included studies. We aimed to present an explicit picture of the capacity building programs on the programs’ dimensions for which information is collected from multiple studies. For this purpose, we outlined the programs’ objectives, learning contents, learning methods, evaluation of capacity building outcomes, duration of programs, and participant selection methods of the included studies. Within the dimensions of programs (see ‎Data Extraction section), we also applied classification that revolves around program objectives. Therefore, the classification of program objectives provided other dimensions with a basis for classification. We also reported overall numbers of studies selected for inclusion in the review.

###  Qualitative Study 

 The qualitative study was conducted once the review results were synthesized. In the qualitative study, we assembled an expert panel to discuss the validity of policy options for local contexts. We sent a policy brief, developed based on the review results, to the members of the expert panel, two weeks before the due date of the panel. We began the panel by presenting the results of the literature review in terms of objectives, contents, and methods, and duration of capacity building programs. Experts discussed the implementation challenges of policy options proposed in the policy brief.

####  Participant Selection and Discussion Guides 

 The participants of the expert panel were selected based on a theoretical sampling.^27^ This sampling method was used since participants should have an in-depth understanding of and be experienced in the topic of interest. We invited 15 experts who had several years of experience in developing and implementing capacity building programs and/or the experience of management of healthcare organizations. The expert panel was conducted at the Knowledge Utilization Center of Tehran University of Medical Sciences in August 2019. A policy brief containing a list of policy options was sent to experts beforehand. The policy dialogue started with 20 minutes presentation of literature review results and continued with questions on the feasibility, barriers, and facilitators of policy options developed through the review. All discussions were recorded and verbatim transcribed.

####  Content Analysis 

 We conducted content analysis in four steps: de-contextualization, recontextualization, categorization, and compilation.^28^ Decontextualization refers to obtaining the sense of the whole transcribed text and identifying meaning units ie, codes. We analyzed the text around the main categories that we aimed to elaborate on the knowledge elicited from the expert panel. These categories comprised of the barriers, facilitators, and feasible interventions for implementing the capacity building program for the Iranian health system. For recontextualization, we went through the transcribed text to assure that all the dimensions of the text have been converted into codes. Categorization refers to condensing the text, identifying sub-categories, categories, and themes. The fourth step refers to the process of writing up. To maintain credibility, we conducted a member check and two researchers analyzed data.

## Results

 Study results are presented in two separate subsections: the literature review and the qualitative study.

###  Review Results 

 We included 11 articles, which contained three programs in Canada, three programs in Nigeria, two programs in the United States, one program per country in the United Kingdom, Fiji, and Malawi, one program was shared between Mexico and Nicaragua and one was shared between South Africa and Cameroon. Some articles included more than one program. Details of data extracted from included studies are given in Table S1 (see Supplementary file 1).

####  Programs’ Objectives

 We distinguished objectives into three types (Table 2): single-skill development, personal/professional development, and organizational development (see Supplementary file 1 for details). Programs with single-skill objectives were focused on developing skills for EIHP.^29-31^ The core of single-skill programs rests on creating the capacity to acquire, understand, assess, and apply research evidence. Personal/professional development programs aim to develop the whole person as a professional decision-maker rather than only developing a single skill. In these programs, leadership development is the main objective.^32,33^

**Table 2:** Summary of Review Results for Capacity Building

**Aspects**	**Summary of Findings **
Programs’ objectives	Three types of objectives were identified: (a) single-skill development programs focused on developing skills to acquire, understand, assess, and apply research evidence. (b) personal/professional development programs aimed at developing the whole person as a professional decision-maker (leadership development) rather than only developing a single skill. (c) organizational development aimed at developing decision-making capacity in healthcare organizations through enhancing the decision-makers’ capacity and providing opportunities to exercise increased knowledge and skills of EIHP.
Target groups	Strategic managers, mid-level managers, policy-makers, policy advisers in the health sector or non-profit organizations.
Contents of programs	Programs’ contents were aligned with the programs’ objectives. (a) single-skill programs consisted of seven technical skills for EIHP such as skills to access, identify, evaluate, and use research findings. (b) personal/professional development programs additionally included materials for enhancing soft skills such as networking, thinking styles, partnership building, and getting an endorsement from provider organizations for policy briefs and review findings. (c) in addition to the materials mentioned for single-skill and professional development programs, organizational development programs comprised contents on the context of a healthcare organization, culture change, change management, and leadership skills so that participants learn to understand organizations, mobilize resources, and initiate and sustain changes.
Training methods	Lectures, workshops, mentorship, and participation in research activities were common methods of training. Most programs presented online or face-to-face lectures or workshops to increase theoretical knowledge of EIHP. Programs also used exercises eg, writing policy briefs, research summaries, reviews to apply learning in practice. Mentorship methods using information technologies (emails, web, and telephone) were to support participants in applying skills in practice.
Evaluation of program results	The evaluation of programs has been conducted at the individual, program, and organizational levels. In most of the quantitative analysis, the assessment was based on the pre-and post-self-assessment tests. Other methods of evaluation included document analysis, interviews, and focus group discussion. Few studies reported the results of the evaluation at the organizational level.
Duration of programs	The duration of the program varies from a couple of days to short-term, medium-term (12 months), and long-term (two years).
Participant selection	Participant selection was not performed in most programs. In long-term programs, committees selected participants based on clear eligibility criteria or participants were invited based on managerial positions.

Abbreviation: EIHP, evidence-informed health policy-making.

 Organizational development programs aim to enhance decision-making in healthcare organizations by enhancing the capacity of decision-makers and providing them with opportunities to exercise increased knowledge and skills of EIHP. Three programs ‘Executive Training for Research Application (EXTRA), ‘Swift, Efficient Application of Research in Community Health’ (SEARCH), and ‘Service Delivery and Organization’ (SDO) programs mentioned objectives to enhance personal/professional development. These programs address skills for EIHP and skills for leadership, change management, and understanding the context of the healthcare organization. These programs also provide participants with opportunities to execute skills for solving real-life problems of their host organizations. In the EXTRA and SEARCH programs, participants used their acquired skills and knowledge to solve organizational or policy issues pertinent to the state or provincial health systems.

####  Target Groups 

 Programs considered various target groups including strategic managers,^34^ mid-level managers, policy-makers, and policy advisers in the health sector or non-profit organizations. Moreover, healthcare delivery managers were targeted by some programs. In Canada, EXTRA and SEARCH programs focused on strategic healthcare management positions at the provincial and/or national levels. In the United Kingdom, the SDO program aimed at increasing research application among mid-level managers with potential for growth to top-level management positions.^35^ In the United States, public health professionals were trained for EIHP.^29,36^ In developing countries, capacity building programs were provided to a wide variety of decision-makers, policy advisers, and operational managers of healthcare delivery organizations.^31-33^

####  Contents of Programs 

 Programs’ contents were essentially consistent with the programs’ objectives. The single-skill programs mostly consisted of seven technical skills for EIHP such as skills to access, identify, evaluate, and use research findings.^29-31,37^ Some programs used standard tools, eg, Supporting the Use of Research Evidence (SURE), Grading of Recommendations, Assessment, Development, and Evaluations (GRADE), A Measurement Tool to Assess systematic Reviews (AMSTAR), for training seven technical skills of EIHP.^33^ Contents on how to synthesize evidence, eg, research summary, were included in certain single-skill programs. Personal/professional development programs additionally included materials for increasing soft skills such as networking, thinking styles, partnership building, and getting an endorsement from provider organizations for policy briefs and review findings.^32,33^ In addition to the materials mentioned for single-skill and professional development programs, organizational development programs comprised contents on the context of the healthcare organization, culture change, change management, and leadership skills so that participants learn to understand organizations, mobilize resources, and initiate and sustain changes based on research evidence that was collected during research projects.^34^

 Contents for research skills eg, rapid review, systematic review, empirical research, and problem-solving skills were intensive in EXTRA and SEARCH programs. The UK fellowship program however included no pre-specified contents as it meant to be a tailored training. The contents of this program were defined based on the specific needs of participants.^35^

 The most comprehensive program was EXTRA with six modules.^34^ Module 1 promotes the use of research evidence in healthcare organizations through learning to deal with policy factors and policy context. Module 2 increases research literacy. Module 3 teaches skills to use research evidence in healthcare organizations. Module 4 focuses on learning individual leadership skills as well as the way that organizational culture and politics influence the conception and use of evidence. Module 5 considers skills, strategies, and resources to monitor and sustain long-term change in organizations. And module 6 provides participants with opportunities to present the value of their research.

####  Training Methods

 Three common training methods were: lectures and workshops, conducting or participating in research activities, and goal-oriented mentoring. Almost all programs included online or face-to-face lectures or workshops to increase the theoretical knowledge on the key skills of EIHP. Programs then continued with research activities to apply acquired knowledge to solve or to exercise solving real-life problems through writing policy briefs,^38^ research summaries,^33^ literature reviews,^37^ and research projects.^34^ Programs used various supporting methods to assist participants in applying skills to solve real-life problems. Mentorship methods were used to support participants in applying skills in practice.^30,32,34,37^ The majority of programs provided some types of mentoring supports for participants.^2,30,32^ However, the duration of mentoring varied between programs. Programs also reported using information technologies such as emails, web tools as well as telephone calls to support participants. Most programs used tailored training, which defines educational contents according to the requirements of participants.^30,34,38^

 In SDO and EXTRA programs, participants take part in a research process from writing a research proposal to data collection, data analysis, and the presentation of findings. The difference between these two programs was that in EXTRA, participants write and conduct research projects with the support of mentors, but in the UK’s fellowship, participants take part in a university research project and learn by being involved in the project. It is worth noting that participants in SDO, EXTRA, and SEARCH programs work on real-life problems of their organizations.

####  Evaluation of Program Results 

 The evaluation of programs has been conducted at the individual, program, and organizational levels. The evaluation of the program’s results relied on both quantitative and qualitative research methods. In most of the quantitative analysis, the assessment was based on the pre-and post-self-assessment tests.^31,34^ Depending on the type of program, other methods of evaluation included document analysis, interviews, and focus group discussion.^37^

 The evaluation at an individual level assesses increases in knowledge and enhanced perceived skills to apply EIHP tools in practice. At least three programs reported positive impacts on knowledge and perceived skills at the individual level.^31,32,39^ For instance, in the EXTRA program, a cohort of participants consisting of health service executives, physicians, and nurses reported the positive impact of the program at the individual level. This program improved research literacy by 55% and the ability to promote the use of research evidence in an organization by 70%.^34^

 Few studies reported the results of the evaluation at the organizational level. The impacts of the SEARCH program at the organizational level were evaluated using qualitative methods,^34^ which showed a modest positive impact of the program. A study assessing the impact of training to apply EIHP in public health reported that 45% of participants think that training increased evidence-based public health in their organizations.^38^ Moreover, programs were evaluated regarding practical improvement in skills related to EIHP such as writing policy briefs,^31,38^ conducting a systematic review, and doing applied research.^34^ The frequency of referring to course materials later on in planning or evaluating public health operations was also used as an indicator of program impact at the organizational level.^36^

####  Duration

 Depending on the program objectives, the duration of the program varies. The EXTRA program had a short-term or midterm duration for professional/personal development objectives and a long-term duration for the health system development. The SEARCH program took 2 years. The SDO fellowship was a 12-month full-time program or if part-time, then equal to 12 months. Most other programs, given being focused on enhancing single skills, took some days.^31,32,36^

####  Participant Selection

 Participant selection was not performed in most programs with explicit criteria or a long-term perspective, except in EXTRA, SEARCH,^34^ and SDO programs.^39^ Single skill-focused programs were running for a short period of time, at most a couple of weeks. Participants of these programs eg, decisionmakers or field managers were generally invited to participate in the training, regardless of their interest, time, or motivation. In contrast, EXTRA, SEARCH and SDO required long term (at minimum 1 year) efforts, thus participants were selected thoughtfully considering their interest, time, and potential. In the SEARCH program, a committee at the provincial level, including a top policy-maker, selected program participants. SDO fellowship had clear eligibility criteria for candidates. In SDO, individuals with managerial positions and those at mid-level but highly motivated, with great potential to promote to a senior management level, or infigdividuals with a willingness to participate in the research were selected for the program.

####  Policy Options 

 Based on the review, we developed the following policy options.

- In the short term, the Iranian Ministry of Health and Medical Education (MoHME), as the main actor of EIHP, develops and implements personal/professional development programs to create a critical mass (eg, a large enough cohort of policy-makers with EIHP knowledge and skills who can qualitatively transforms institutional culture, norms, and values of decision-making for applying evidence to policy).^40^
- In the long-term, MoHME develops and implements capacity building programs to enhance the performance of healthcare organizations through a change in organizational culture towards EIHP.
- For capacity building programs, MoHME uses current national healthcare management training programs (eg, Training of Trainers for Healthcare and Hospital Management).
- MoHME takes steps to break the causal chain or to change contextual factors that blur the needs for EIHP skills, training programs, and to apply EIHP in real-life practice.

###  Qualitative Study Results 

 The expert panel affirmed the value and use of research for both policy and practice. They reflected on the design of the capacity building programs and barriers and facilitators. Informants also suggested interventions to tackle barriers or address constraints to promote EIHP.

####  Design of Capacity Building Programs 

 Experts made a distinction between the characteristics of capacity building programs before- and after-assignment of individuals to management or policy-making positions. They believe that training programs through residency sessions, workshops, and mentoring could be effective before assigning individuals to management positions and after assignment, those methods cannot change the behaviors of decision-makers to apply EIHP. They suggested practical training such as project-based learning or learning by doing for after assignment to a decision-making position. Regarding this, a quote from one experienced trainer and healthcare provider follows:

 “*Training such as residency sessions, workshops, and mentoring are effective methods before an assignment and could prepare a person for finding, analyzing, and using research evidence for a future managerial or policy-making position.… Training after assignment [include training] such as project-based training systems ‘learning by doing’ and giving adequate training during occupying a position. [However,] in Iran since person receives organizational authority and power from elsewhere [than the legitimate sources of power], this way of training would be feasible on paper and in practice would not find a possibility for implementation*.*” *

 Codes on making a distinction between capacity building programs for policy-makers versus those who identify policy options, and between healthcare managers versus policy-makers emerged in several places in the transcribed text. The qualifications, roles, and authorities of these groups are different; consequently, there should be different methods and the contents for their capacity building programs. Particularly, managers are focused on organizational issues; whereas, policy-makers are dealing with systems that are more complex than organizations. Tailoring capacity building programs to the educational needs, requirements and contingencies of these target groups were emphasized by experts.

 Informants also emphasized training to create capacity for problem-solving in healthcare organizations. Given this, one participant was concerned that the proposed programs are unlikely to increase capacity of policy-makers. For instance, she remarked that the proposed policy options for capacity building programs and the contents proposed are unlikely to address the needs of the policy-making bodies at the MoHME and the Health Commission of the Iranian Parliament.

 Some of the suggestions pointed out to the experiences of previous programs. The selection of trainees was a key issue in earlier capacity building programs, as some trainees were at the end of their career in the public sector, and they were about to leave the system without any return on the training.

 Experts also pointed other types of conditions before the implementation of new capacity building programs. One expert mentioned that we have already an adequate number of management training programs but evaluating previous programs before implementing new ones is more important than just launching a new one. It was emphasized that prior capacity building programs should be evaluated, and the evaluation should be based on clear, transparent, and specific criteria.

 Having learned about the capacity building programs, experts suggested several interventions to help facilitate the implementation of EIHP and reinforce the capacity building programs. These interventions considered prerequisites and supportive contexts for EIHP, addressing capacity building at the team level rather than individual level, and learning from the past capacity building programs.

####  Barriers 

 Barriers include accountability and responsiveness, conflict of interests in the context of the healthcare system, and organizational structures and processes.

####  a. Context of the Healthcare System 

 Based on the experts’ views, the style of decision-making, which is largely a factor in the context of the Iranian health system, does not make any commitment to the use of evidence. This style, like an established culture, forms decisions and determines the quality of decisions. The style is shaped by the lack of accountability and responsiveness in the whole context of the health system. Informants state that managers should be kept responsible for their decisions. For example, one informant who has significant experience in training and strategic management stated that responsiveness should be embedded in the structure of organizations and the health system and also be demanded by employees at the bottom of the organizational hierarchy. The sense of responsibility encourages decision-makers to follow training and to make evidence-based decisions.

*“The decision-making style in Iran does not commit to the use of evidence. Decision-makers are not questioned about the base or motivation of their decisions*.* If managers were kept responsible for their performance, [they] will pursue training and making decisions based on evidence… Demanding [responsiveness] should begin from the bottom [of organizations] and keep governing systems responsible. If responsiveness increases, the manager commits to behaving based on evidence. The structure should be designed in a manner that keeps the manager responsive at every moment. For being responsive, the manager needs to involve others and as a result, evidence would be collected, [in turn] leading to collective logical and rational decisions*”(Policy-maker and trainer).

 Several experts emphasized understanding what real problems that prohibit EIHP. According to the experts, managers know existing evidence and know how to use it, but one real problem, among others, is the conflict of interest. As a result, managers look for pieces of evidence that support their decisions. Below, a quote from a trainer follows:

*“Research has shown that in most cases, policy-makers know the evidence and know that they should use [evidence] but do not use it. Managers are not looking to find the right evidence, but [they are] up to make up evidence to justify what they have in their mind. Thus, the problem is not knowing [evidence] or capacity building [programs] only. Other issues are also involved such as the conflict of interests.” *

 The expert panel also provided recommendations to tackle the contextual challenges. One key decision-maker with several years of experience remarked that prerequisites in the context of health systems or in the structure of organizations need to be present to ensure that the context is receptive to individuals who have developed capacity for EIHP. Preparing the context requires resolving critical issues, notably the conflict of interests, in the context and structure;

*“The capacity building programs could be effective contingent on satisfying prerequisites and meeting requirements that rest in the context or structure of healthcare organization; otherwise, in undeveloped organizations, developed individuals make no difference. Unless certain problems such as the conflict of interest, are resolved in the health system, the path discussed in the meeting would not be possible to be implemented.” *

####  b. Organizational Structures and Processes 

 Organizational structure in terms of management authority, decision support systems, and knowledge management infrastructures were referred to as the barriers in the organizational structures. While participants discussed the chain of causes that hinder EIHP, the structure of healthcare organizations was emphasized as the root cause of the lack of EIHP. As one healthcare provider and trainer stated, the structure acts as a bottleneck that limits the overall use of evidence.

*“In practice, a chain of causes leads to the current situation [of the lack of EIHP in Iran]; therefore, our conversation today about training and empowerment may have other main root cause as the main barrier or driver [of EIHP]. One of these main causes is the structure of organizations. Therefore, opinions that are offered [in this meeting] may independently and individually be right, but when run in the context of an organization, somewhere stop and are trapped, and out of this discussion that intended solution may not be immediately obtained and needs integration [into the context of organization].” *

 One informant remarked that the management authority to make key decisions is limited; policies are determined at a higher level. On the other hand, there was a counterargument that *“having a greater authority for decision-making does not have a very clear relationship with acting based on evidence, it may even lead to making inappropriate decisions via this inappropriate authority*.*”*

 Management tenure is so short, which prohibits focusing on main solutions such as EIHP. Furthermore, there is a high expectation from managers to deliver results, at the same time, no decision support system exists to assist them in handling a pile of tasks. Knowledge management and the free circulation of information are not yet institutionalized in our organizations. In this situation, healthcare managers tend to embark on short-run projects.

 Participants stated that managers have no time to think, thus it is necessary to support managers through think tanks, advisors, and management teams. In addition to decision-makers, individuals whose job is to develop or to identify policy options, need capacity building programs. Furthermore, management teams and not only managers need training.

 Experts also made statements about the process to fill in management positions. The process for selecting, assigning, and changing managers should be updated. One participant cast doubt on the efficacy of capacity building programs to empower policy-makers, as they are not selected based on their capabilities.

####  Facilitators 

 Facilitators that were identified are related to the current infrastructures, rules, and regulations that promote capacity building programs. One informant underscored the capacity of current healthcare management graduates, which are almost abandoned in the health system. The capacity of the educational systems of the country can also be used to deliver capacity building programs.

 There was a remark that decision-makers and managers have a ‘Life Course View’ indicating that they prefer to leave a legacy behind. This view may reinforce the capacity building programs if the managers are convinced that they could even have a more significant legacy by using evidence-informed decision-making tools and techniques.

###  Mixed Findings

**Figure 2:**
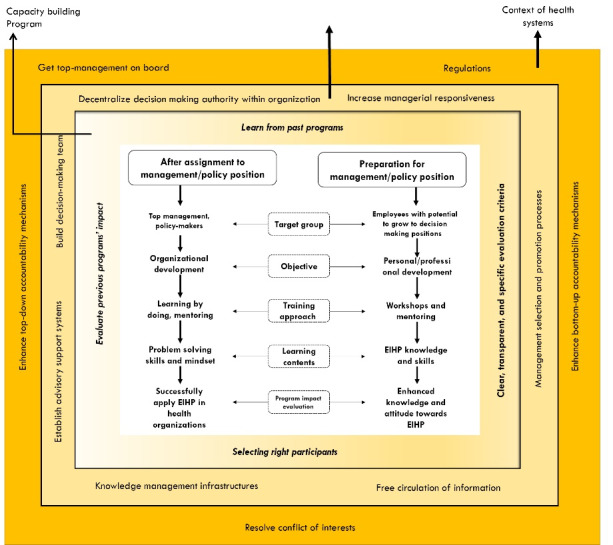
Framework Incorporating Interventions at the Program Level, Organizational Level, and Health System Context for Increasing The Capacity of Health Policy-Makers. Abbreviation: EIHP, evidence-informed health policy-making.

 We mixed findings from the literature review and the expert panel in Figure 2 to construct a framework to increase the capacity of policy-makers to apply EIHP in Iran.

 We configured policy options at three levels: program, organizational, and the context of the health system. Starting with the program level, we envisaged two distinctive lines; designing capacity building programs for before-assignment and programs for after-assignment to management or policy positions. These two lines have distinctive target groups, objectives, training methods, contents, and impacts. The surrounding issues of both lines regard the way that programs are designed and considerations that are taken into account for designing programs. These considerations include scrutinizing previous programs, lessons learned from past programs, and developing transparent and specific criteria for the evaluation of programs. Furthermore, the right participants should be selected for programs, given training is like an investment that needs to have a return.

 At the second level, the policy options address factors in the structure of organizations. This level, on one hand, suggests increasing management authority, and on the other, it suggests increasing the management responsiveness to make a balance between authority and responsibility. To support management authority for making a decision based on evidence, it suggests establishing advisory systems and information infrastructures and building decision-making teams. Interventions at this level also consider selecting the right people for management positions and free circulation of information.

 At the third level, there are contextual factors that also impede the impact of capacity building programs. Resolving the conflict of interest for decision-making positions is a key factor. Change in the context may regard strengthening two lines of accountability, through top-down mechanisms and bottom-up mechanisms.

## Discussion

 The majority of reviewed programs were focused on developing a single skill rather than on professional development or organizational development. Programs that were focused on professional or organizational development were delivered in resource-rich countries such as Canada. By moving from single-skill programs towards the personal/professional and then organizational development programs, learning contents also include materials on soft skills, decision-making mindset, networking and partnerships, change management, and leadership skills to execute and sustain changes.

 The evaluation of programs at the individual level showed positive impacts of the programs on the knowledge and confidence of participants. Few studies assessed the impacts of programs at an organizational level. The evaluation of programs’ impacts at this level assessed the spread of knowledge and attitude of EIHP to other members in organizations for which very little evidence is generated.^3^ When participants held the central role and/or leadership positions in their organizations, the change of attitude towards EIHP was facilitated.^2^ It is worth noting that, there is no report for the evaluation of capacity building programs at the health system level, which indicates the lack of research or the presence of publication biases.^34^

 The literature review showed that dedicated programs attract management attention to the program and make them more committed to participating.^3^ Reinforcing this theme, the participants of the expert panel also emphasized dedicated programs and made a distinction between the characteristics of programs before versus after assigning to management positions. We incorporated this as the core of our framework in Figure 2. Experts believed that objectives, learning materials and contents, and training methods of programs for these conditions should be different. Capacity building programs that concern enhancing the knowledge or improving EIHP skills would have a little impact after the time that a person is assigned to a decision-making position. Programs with the personal development objective could be more effective before assigning individuals to management positions and will prepare program attendees for finding, analyzing and using research evidence. Learning skills of EIHP require tremendous time and energy. Due to work pressure, management positions can hardly meet such requirements.

 After assignment to management positions, problem-solving skills are more important as they accommodate the real needs of organizations. When individuals hold management positions, learning by doing via support from mentors with local knowledge could be an effective method for capacity building.^41^ Problem-solving was a common thread between the review and the expert panel. Programs such as EXTRA and SEARCH, which also intended to impact organizations, were organized around helping program participants to be able to solve real-life problems.

 As given in the framework to increase the capacity of policy-makers on EIHP, efforts to increase the capacity of policy-makers need to prioritize interventions for breaking the chain of causes that leads to inadequate uptake of EIHP. Within the chain of causes, the contextual factors and organizational structure were overrepresented by the expert panel.

 Among contextual factors, informants insisted on the intervention to resolve the widespread conflict of interest in the health system as a prerequisite or even a bottleneck that limits the overall progress of EIHP in Iran. The conflict of interest has been growing in the health sector in the past decades. Several factors give rise to this issue, such as the regulatory capture and mixed responsibility of both policy-making and service provision in a single body ie, MoHME. As a result, many evidence-based reforms find no chance to be implemented, such as family physician programs.^42^ Therefore, in the first place, conflict of interest should be resolved, otherwise, interventions on other factors would not bring about desired outcomes.^42,43^ It is however worth noting that interventions to resolve conflict of interest may take a long path or may never succeed.

 As mentioned in the expert panel, the context of the Iranian healthcare system is not aligned with EIHP. As a result, little sense of responsibility exists to support learning or to motivate pursuing training programs to improve the quality of decisions. An accountability mechanism, either top-down, as a management control mechanism, or bottom-up, as a public demand mechanism was recommended to make decision-makers more accountable.

 Weak accountability mechanisms are accompanied by poor advisory or decision support systems and knowledge management infrastructures for management positions with a pile of unimportant tasks to do and staffing shortage.^3^ Therefore, investments in advanced decision support systems and knowledge management infrastructures are essential to ensure facilitated access to evidence.

 Another closely related intervention is that to support decision-makers by building a management team or decision-making team and training the teams. However, these arrangements could be developed through organizational processes instead of waiting for reforming organizational structures. Jacobs and colleagues reported that the team as a whole should be able to practice the EIHP process.^36^ Another study reported that training a team or group of individuals from the same organization had positive organizational impacts by spreading the attitude of EIHP in the organization.^3^ This however requires a certain degree of decentralization for which there were two different views among the panellists; one refers to limited managerial authority, another indicates that practicing EIHP has nothing to do with authority and that with less-developed decision-makers, a larger authority leads to even more inappropriate decisions.

 Even though most of the suggested interventions on their own make sense, implementing them might be time-consuming. Aligning the context of the health system and structure of healthcare organizations with EIHP may take a long time. Therefore, instead of waiting for fully ready context and organizations for EIHP, decision-makers should learn to work in the existing context and make and sustain changes. In EXTRA and SEARCH programs, participants learned skills to understand the complex context of health organizations and to create and sustain improvements through change management and leadership skills in the current contexts.^2,34^

 This study faced several limitations. First, while experts shed great light on the structural and contextual factors that are pivotal for effective capacity building programs, we provided experts with no review of structural or contextual factors in the literature. Other subprojects of the Iran EIHP study synthesize policy options to address dimensions of structures of healthcare organizations. Second, our search strategies may have overlooked certain relevant articles that present training for research skills or problem-solving skills among decision-makers. In other words, we likely did not identify a representative sample of the literature in our review. To deal with this limitation, we searched the Department of Health in Australia, Canada, and the United kingdom for the pull efforts that were presented in annual reports and so forth. These departments were selected based on consultation with experts in evidence-informed decision-making. The search in these departments was not as systematic as the search strategy applied to the databases.

## Conclusion

 The positive impacts of capacity building programs on the knowledge and attitude of policy-makers are reported. However, a long path remains to accomplish the impacts of programs at policy-maker behaviours, healthcare organizations, and health systems. Paving this path requires the rearrangement of capacity building programs. We followed various threads from the literature review through to the expert panel so to develop our framework and strategies for capacity building programs in Iran. These threads included, but not limited to, building and training a management team instead of only training managers, focusing the training on problem-solving skills, and designing dedicated programs based on the needs of target groups. Based on the proposed framework in Figure 2, various interventions at the program level, organizational level, and health system context can be done to make the overall health system receptive to EIHP and capacity building programs. Notably, competing interests, as a bottleneck factor, need to be resolved, decision-makers should be made more accountable, and healthcare organizations need to provide more knowledge management infrastructures and advisory staffs to support decision-makers to apply EIHP.

## Acknowledgements

 We are grateful to all who contributed to conducting this research.

## Ethical issues

 This research was approved by the ethics committees of Tehran University of Medical Sciences (IR.TUMS.VCR.REC.96.02.159.35954) and the National Institute for Medical Research Development (IR.NIMAD.REC.1397.476).

## Competing interests

 Authors declare that they have no competing interests.

## Authors’ contributions

 Conception and design: MM, HSS, BY, and RM. Acquisition of data: MM, SN, RM, JSK, SHGH, RT, HD, LD, HSS, and BY. Analysis and interpretation of data: MM and RM. Drafting of the manuscript: MM. Critical revision of the manuscript for important intellectual content: RM, HSS, EE, and LD. Obtaining funding: RM. Administrative, technical, or material support: SN. Supervision: RM.

## Funding

 This work was supported by the Deputy for Research and Technology at the Iranian Ministry of Health and Medical Education (Grant Number 700.1953) and Iranian National Institute for Medical Research Development (NIMAD) (Grant Number 958431). Funders had no role in any part of the work including design and conduct of the study, data collection, data management, data analysis and interpretation, preparation, review and approval of the manuscript.

## Supplementary files

Supplementary file 1 contains Table S1.
